# Factors associated with early sexual initiation among preparatory and high school youths in Woldia town, northeast Ethiopia: a cross-sectional study

**DOI:** 10.1186/s12889-019-6682-8

**Published:** 2019-04-04

**Authors:** Eskeziaw Abebe Kassahun, Abebaw Addis Gelagay, Achenef Asmamaw Muche, Amanuel Addisu Dessie, Belayneh Ayanaw Kassie

**Affiliations:** 1 Department of Midwifery, Faculty of Health Sciences, Woldia University, Woldia, Ethiopia; 2 Departments of Reproductive Health, Institute of Public Health, College of Medicine and Health Sciences, University of Gondar, Gondar, Ethiopia; 3 Department of Epidemiology and Biostatistics, Institute of Public Health, College of Medicine and Health Sciences, University of Gondar, Gondar, Ethiopia; 4 Department of Public Health, Faculty of Health Sciences, Woldia University, Woldia, Ethiopia; 5 ICAP/NEPI project, College of Medicine and Health Sciences, University of Gondar, Gondar, Ethiopia

**Keywords:** Early sexual initiation, Youth, Students, Woldia, Ethiopia

## Abstract

**Background:**

Early initiation of sexual activity affects the sexual and reproductive health of the young population. The youth are at a high risk of risky sexual behaviours, including multiple partners and inconsistent condom use. There has been limited research on the level and determinants of early sexual initiation in Woldia town. Thus, this study aimed to assess the prevalence of early sexual initiation and associated factors among preparatory and high school students in Woldia town, northeast Ethiopia.

**Methods:**

An institution based cross-sectional study was conducted on 723 students selected by the simple random sampling technique on March 7, 2016. A pre-tested and structured self-administered questionnaire was used for data collection. Descriptive statistics, bivariate and multivariable logistic regression were computed. Adjusted odds ratio (AOR) with a 95% confidence interval (CI) was calculated to examine the strength of association. In the multivariable analysis, a *p*-value < 0.05 was considered as statistically significant.

**Result:**

The prevalence of early sexual initiation among preparatory and high school students in Woldia town was 18.4% (95% CI:15.50,21.30%). Not attending religious programs (AOR = 3.2, 95% CI:1.84,5.44), peer pressure (AOR = 1.9, 95% CI:1.14,3.25), cigarette smoking (AOR = 2.3, 95% CI:1.06,4.85), poor parental monitoring (AOR = 2.8, 95% CI:1.77,4.53), and exposure to pornographic materials (AOR = 2.7, 95% CI:1.68,4.40) were significantly associated with early sexual initiation.

**Conclusion:**

A large number of students initiated sexual activity at an early age. The practiced is associated with sexual and reproductive health problems. Therefore, raising awareness of students about the risk factors for and implication of early sexual initiation through teachers, religious leaders, and parents is highly recommended.

**Electronic supplementary material:**

The online version of this article (10.1186/s12889-019-6682-8) contains supplementary material, which is available to authorized users.

## Background

The World Health Organization (WHO) defines youth as a group of people between the ages of 15 to 24 years which is characterized by a rapid progression from the appearance of secondary sexual characteristics to sexual and reproductive maturation. This period is also the time when youth face many challenges. The decisions they make during this vulnerable period can impact the quality and length of their life [1, 2].

Early initiation of sex exposes young people to many sexual and reproductive health problems. Youth who begin early sexual activity are more likely to practice risky sexual behaviours, such as multiple sexual partners and incorrect or inconsistent condom use. As a result, they increase the risk of sexually transmitted infection (STIs), including HIV/AIDS, unwanted pregnancy, unsafe abortion, early childbirth, and psychosocial problems. These problems are the greatest threats to health and wellbeing of the youth [3, 4].

According to the 2016 Ethiopian Demographic and Health Survey (EDHS) report, 24 and 62% of women initiated sex before the ages of 15 and 18 years, respectively [5].

Globally, adolescent and youth represent a growing share of people living with HIV/AIDS. Each year, 670,000 youth are infected with HIV. Of these, 250,000 are adolescents [6], and 16 million of them give birth [7].

In Africa, a high proportion of unsafe abortions are among adolescents and young women. About 60% of the unsafe abortions occur among women under 25 years [8].

In Ethiopia, unwanted pregnancy among adolescent and the youth is the major sexual and reproductive health challenge. Fifty-four percent of women under the age of 15 years and 37% of youth aged 20–24 years have an unwanted pregnancies [9].

Numerous socio-demographic, economic, behavioral, and parental characteristics, including age [10–12], sex [13–18], residence [12, 17], parental monitoring [11, 19, 20], behavioral factors [13, 15, 16, 18, 19] and peer pressure [11, 12] were significantly aassociated with early sexual initiation.

There has been only limited information on the magnitude of early sexual initiation and associated factors among the youth in northeast Ethiopia, particularly in the study area. Understanding and identifying factors associated with early sexual initiation are essential for developing effective policies and strategies to reduce the adverse consequences of early sexual initiation. Therefore, this study aimed to assess the prevalence and risk factors of early sexual initiation among preparatory and high school students in Woldia town, northeast Ethiopia.

## Methods

### Study setting and period

An institution based cross-sectional study was conducted on March 7, 2016 in Woldia, northeast Ethiopia. Woldia is found 520 km from Addis Ababa, the capital of Ethiopia. According to the 2016 Woldia town administrative report, the estimated population was 75,446, of which 50.6% were males [21]. In the town, there was one preparatory and three high schools with a total of with a total of 4671 students. There were 87 sections, of these 59 sections were in high schools.

### Sample size and sampling procedures

All regular preparatory and high school students in the town during the study were included. The required sample size was calculated using the single population proportion formula by considering a 95% confidence interval (CI), 3% margin of error(d), and the proportion(p) of early sexual debut 19% from the previous study in Shire-Endasellassie town, Tigray region [22]. Considering a 10% non-response rate, the final sample was 723 students. The simple random sampling technique with proportional allocation to each grade level was applied to reach participants. Thus, 269, 177, 143 and 129 students were selected from grade 9, 10, 11, and 12, respectively.

### Data collection tool and techniques

The data were collected using a structured self-administered questionnaire (Additional file 1). The questionnaire was first prepared in English and translated to Amharic (the local language) and back-translated into English by language experts to ensure an accurate translation. Ten data collectors were selected from Woldia University 4th year Midwifery students. Interviewers received 2 days intensive training prior to data collection. The questionnaire was pretested on 37 students in Kobo preparatory and high school out of the study area. The purpose and objectives of the study were clearly explained to participants before data collection. No personal identifiers were used on data collection forms, and teachers were requested to leave the classroom. To maintain privacy, seats were arranged far apart and copies were collected on the ballot.

### Measurements

#### Age at sexual initiation

The age at which students had penetrative sexual intercourse from the first time.

#### Early sexual initiation

Having sexual practice before the age of 18 years.

#### Sexually active

Students who claimed to have engaged in sexual activity at least once prior to the study.

#### Peer pressure

Pressure from friends to have sexual intercourse.

#### Pornographic materials

Refers to newspapers, magazines, books, photographs, movies, the internet intended to sexually arouse the viewer.

#### Religious participation

Student participation in religious programs.

### Assessment of wealth index and parental monitoring

Household wealth index adopted and developed from EDHS 2011 [23] was assessed using Principal Component Analysis (PCA) by considering the numbers and kinds of goods ranging from television to car, agricultural land ownership, quantity of cereal products, livestock and housing condition, such as flooring materials, household cooking material and place. Firstly, variables were coded between 0 and 1; then, the variables were entered and analyzed using PCA. Variables which had communality values greater than 0.5 were considered to produce factor scores. Next, the produced factor scores were computed to produce common factor scores. Finally, common factor scores were summed and categorized into quintile as lowest, second, middle, fourth, and highest. Similarly, paternal monitoring was assessed using a four-item Likert scale which was adapted and developed from previous studies [24, 25]. The items used to assess parental monitoring were ‘How often do your parents try to know who are your friends’, ‘Do your parents know where you are outside of home or school’ and ‘Do your parents know with whom you are outside home or school’. The responses ranged from ‘never’ (coded as 1) to ‘always’ (coded as 4). Finally, via PCA, it was classified as ‘poor’ and ‘good’ parental monitoring.

### Assessment of school connectedness and knowledge of HIV/AIDS

Student school connectedness was assessed using a five-item Likert scale adopted and developed from a previous study [26], such as ‘Do you feel close to people who are at school’, ‘Do you feel happy to be at this school’, ‘Do you feel as if you are a part of this school’, ‘Do school teachers treat students fairly at this school’, and ‘Do you feel safe being at this school’. The responses ranged from ‘strongly disagree’ (coded 1) to ‘strongly agree’ (coded as 5). Using PCA, the scale was classified into quantiles as poor and good school connectedness. In addition, knowledge of students on HIV/AIDS was assessed based on the transmission and prevention methods of HIV/AIDS which was adopted from EDHS 2011 [23], and the questions included condom use, number of sexual partners, awareness on healthy-looking person may have HIV/AIDS, and rejection of the two most common local misconceptions about HIV/AIDS that (HIV/AIDS can be transmitted by mosquito bite and supernatural means). Using PCA, the scale was ranked into ‘poor’ and ‘good’ knowledge.

### Data processing and analysis

After the data collection, the data were checked for completeness, coded manually, and entered into EpiData version 3.1 and then exported to SPSS version 20 for analysis. Descriptive analysis was carried out and presented using texts, tables, and graphs. Bivariate and multivariable logistic regression analysis were done to identify factors associated with early sexual initiation. Variables in the bivariate logistic regression with *p*-value less than 0.2 were fitted into the multivariable logistic regression to control the possible effects of confounders. The enter method was employed. Odds Ratios with their corresponding 95% confidence intervals (CI) was calculated to see the presence and the strength of associations. Model fitness was checked by the Hosmer-Lemeshow goodness of-fit test. In the multivariable analysis, variables with *p*-value < 0.05 were considered as statistically significant.

## Results

### Socio-demographic and economic characteristics of participants

A total of 718 students participated in the study with a response rate of 99.3%. The median age of the participants was 17 (IQR ±1.6) years. Approximately, half (50.7%) of the students were female. The majority (91.8%) were in the age range of 15–19 years. In addition, 59.2% of the students did not take life skills training, and 15.6 and 7.7% had deceased fathers and mother, respectively, (Table 1).

**Table 1:** Socio-demographic characteristics of preparatory and high school students, Woldia, northeast Ethiopia 2016(*n* = 718)

Variables	Frequency	Percentage
Sex		
Female	364	50.7
Male	354	49.3
Age		
15–19	659	91.8
20–24	59	8.2
Marital status		
Single	714	99.4
Married	4	0.6
Educational status		
Grade 9	269	37.5
Grade 10	177	24.7
Grade 11	143	19.9
Grade 12	129	18.0
Religion		
Orthodox Christian	517	72.0
Muslim	182	25.3
Others^a^	19	2.7
Religious participation		
Yes	628	87.5
No	90	12.5
Frequency of religious participation (*n* = 628)		
Every day	231	36.8
Once a week	289	46.0
Once a month	52	8.3
Once a year	40	6.4
Others^b^	16	2.5
Living with		
Both parents	388	51.3
Mother only	109	15.2
Father only	24	3.3
Friends	21	2.9
Relative	133	18.5
Living alone	42	5.8
Grandparents	21	2.9
Current Fathers’ educational status (*n* = 606)		
Unable to write and read	60	8.4
Able to write and read	239	33.3
Elementary school (1-8th)	127	17.7
High school (9-10th)	22	3.1
Preparatory school (11-12th)	36	5.0
Collage and above	122	17.0
Current Mother’s educational status (*n* = 663)		
Unable to write and read	129	19.5
Able to write and read	195	29.4
Elementary school (1-8th)	124	18.7
High school (9-10th)	71	10.7
Preparatory school (11-12th)	52	7.8
Collage and above	92	13.9
Current Father’s employment status(*n* = 606)		
Civil servant	181	25.2
Private employer	99	13.8
Merchant	115	16.0
Daily laborer /house maid	17	2.4
Farmer	172	24.0
Others^c^	22	3.1
Current Mother’s employment status (*n* = 663)		
House wife	310	46.8
Civil servant employer	121	18.3
Private employer	46	7.0
Merchant	119	18.0
Daily laborer /house maid	16	2.4
Farmer	51	7.7
Household wealth status		
Lowest	143	19.9
Second	258	35.9
Middle	35	4.9
Fourth	142	18.9
Highest	140	18.5

^a^Protestant and catholic, ^b^Participate religious participation more than once in a week, month, and year ^c^Driver, Soldier, Pensioner

### Parental communication and monitoring, school connectedness, and peer pressure

About 46% of students reported that their parents strongly monitored them, and 49.6% said they had good school connectedness. Moreover, 55.3% of the participants discussed at least one sexual and reproductive health issue with their parents. The most common sexual and reproductive health issue which students discussed with their parents was on HIV/AIDS (Table 2).

**Table 2:** Parental communication and monitoring, school connectedness and peer pressure of school students in Woldia, northeast Ethiopia, 2016 (*n* = 718)

Variables	Frequency	Percentage
Parental monitoring		
Poor	329	45.8
Good	389	54.2
School connectedness		
Poor	362	50.4
Good	356	49.6
Parent-youth communication on SRH issue		
Yes	397	55.3
No	321	44.7
Parent–youth communication on (*n* = 397)®		
Puberty and menstruation	175	44.1
Prevention of pregnancy	91	22.9
Relationship with opposite sex	122	30.7
Unwanted pregnancy and abortion	81	20.4
STIs including HIVI/AIDS	205	51.6
Discussion on other issues^a^	6	1.5
Peer pressure to have sex		
Yes	110	15.3
No	608	84.7

®multiple responses ^a^On pregnancy, Rape, Sexual abuse

### Behavioral characteristics

**Fig. 1:**
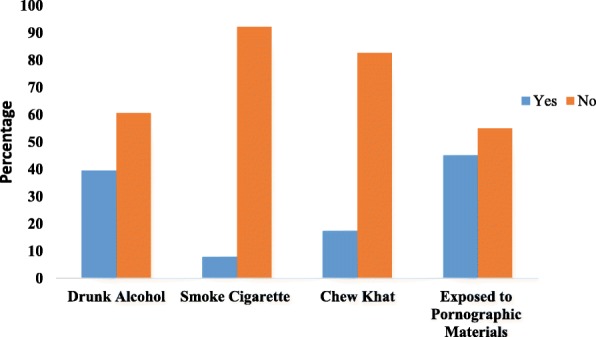
Behavioral characteristics of preparatory and high school students in Woldia town, northeast Ethiopia, 2016

Among the study participants, 39.4, 7.8, 17.3 and 45% reported that they drank alcohol, smoked cigarettes, chewed khat, and were exposed to pornographic materials, respectively, (Fig. 1).

### Prevalence of early sexual initiation

The overall prevalence of early sexual initiation in the study area was 18.4% (95% CI:15.50%, 21. 30%). Out of the participants, 38.3% were sexually active, of whom 55.5% initiated the first sex with their regular boy/girlfriends and 56.7% had two or more sexual partners (Table 3).

**Table 3:** Sexual history of preparatory and high school students in Woldia, northeast Ethiopia, 2016(*n* = 718)

Variable	Frequency	Percentage
Ever had sex		
Yes	275	38.3
No	443	61.7
First sexual partner (*n* = 275)		
Regular boy/girl friend	153	55.5
Casual partner	64	23.3
Husband or wife	3	1.1
Family member	10	3.6
Teacher	27	9.8
Commercial worker	18	6.5
Number of life time sexual partner (*n* = 275)		
One	119	43.3
Two or more	156	56.7
Time of initiation sexual activity (*n* = 718)		
Age less than 18 years	132	18.4
Age 18 years and above	586	81.6

### Factors associated with early sexual initiation

In the bivariate logistic regression analysis, sex, marital status, educational status, attending religious programs, parent-youth discussion, drinking alcohol, smoking cigarettes, chewing khat, smoking shisha, life skills training, parental monitoring, school connectedness, peer pressure, and exposure to pornographic materials were associated with early sexual initiation at *p*-value < 0.2. In the multivariable logistic regression analysis, sex, attending religious programs, peer pressure, smoking cigarettes, parental monitoring, and pornographic materials remained statistically significant and were independently associated with early sexual initiation. The odds of having early sexual initiation were three times (AOR = 3.2; 95% CI: 1.84, 5.44) as high among students who didn’t attend religious programs compared to their counterparts. Similarly, the odds of having early sexual initiation were nearly three times (AOR = 2.8; 95% CI:1.77, 4.53) as high among students who had poor parental monitoring than those who had good parental monitoring. Compared to students who had not been exposed to peer pressure, students who had peer pressure were at increased odds of having early sexual initiation by 90% (AOR = 1.9; 95% CI:1.14, 3.25). Moreover, early sexual initiation was about three times (AOR = 2.7; 95% CI: 1.68,4.40) as high among students exposed to pornographic materials than their counterparts (Table 4).

**Table 4:** Bivariate and multivariable analysis for early sexual initiation among preparatory and high school students in Woldia town, northeast Ethiopia, 2016(*n* = 718)

Variables	Early sexual initiation		COR (95%CI)	*P*-value	AOR (95%CI)
	Yes	No			
Sex					
Female	52	302	1.6(1.11,2.40)	0.012	3.0(1.88,4.76)**
Male	80	284	1.0		
Religious participation					
No	36	54	3.7(2.3, 5.9)	0.000	3.2(1.84,5.44) **
Yes	96	532	1.0		1.0
Peer pressure					
Yes	40	70	3.2(2.05, 5.01)	0.000	1.9(1.14,3.25)*
No	92	516	1.0		1.0
Drinking alcohol					
Yes	80	203	2.9(1.97, 4.28)	0.000	1.5(0.90,2.35)
No	52	383	1.0		1.0
Cigarette smoking					
Yes	26	30	4.6(2.58, 8.00)	0.000	2.3(1.06,4.85) *
No	106	5156	1.0		1.0
Khat Chewing					
Yes	47	77	3.7(2.38, 5.62)	0.000	1.6(0.90,2.78)
No	85	509	1.0		1.0
Smoking shisha					
Yes	12	27	2.1(1.02,4.20)	0.044	0.6(0.26,1.62)
No	120	559	1.0		1.0
Life skills training					
No	92	333	1.8(1.17, 2.62)	0.007	1.4(0.89,2.25)
Yes	40	253	1.0		1.0
Parental monitoring					
Poor parental monitoring	94	235	3.7(2.45,5.58)	0.00	2.8(1.77,4.53)**
Good parental monitoring	38	351	1.0		1.0
School connectedness					
Poor school connected	88	274	2.3(1.53, 3.39)	0.000	1.3(0.83,2.14)
Good school connected	44	312	1.0		1.0
Exposed to pornographic materials					
Yes	169	177	3.7(2.45, 5.56)	0.000	2.7(1.68,4.40) **
No	68	358	1.0		1.0

Note: * (*P*-value < 0.05) and ** (*P*-value < 0.01)

## Discussion

This study was conducted to assess the prevalence and factors associated with early sexual initiation among preparatory and high school students in Woldia town, northeast Ethiopia. The overall prevalence of early sexual initiation among preparatory and high school students in Woldia was 18.4% (95% CI; 15.50, 21.30%). This finding is quite similar to those of other local studies carried out in Shire-Endasellassie town, Tigray region (19%) [18] and Faggeta-Lekoma district, Awi Zone (20.4%) [11], Ethiopia. This finding is lower than reports in Addis Ababa (25.4%) [27] and the EDHS 2016 report (62%) [5]. The difference may be accounted for by time trends. There is an increasing impact of globalization or new technologies that influence the healthy sexual life of students. Furthermore, the possible difference from the EDHS result might be variations of study areas in that the EDHS study was conducted in both urban and rural areas of all regions of Ethiopia which had different socio-cultural characteristics and access to youth reproductive health services. Additionally, in the rural areas of Ethiopia, family arranged marriages at young age are common.

Girls were more likely to initiate sexual intercourse before the age of 18 years than boys. This finding is in line with those of other studies in Ethiopia, such as EDHS 2016 [5], Dessie [28] and Debre-Markose [13]. This might be due to the effect of culture that forces females to marry at young age. Besides, boys have greater access to education than girls.

Religious institutions have been focusing on advising and counseling individuals to delay sexual initiation and discourage sex before marriage, substance use, and multiple sexual partners. Generally, religious institutions play an important role in youth to develop healthy lifestyle. In this study, students who participated in religious education programs were less likely to initiate sex at an early age. The finding is in line with those of other studies done in USA [29], and Addis Ababa, Ethiopia [30].

In addition, students who smoked cigarettes were more likely to start sex at an early age. The same finding was noticed by other studies done in Debre-Markose [13], China [31], and Addis Ababa [30].This might be due to the effect of substance which alters healthy thinking ability of the youth and results in unplanned and unsafe sex.

Students who had good parental monitoring were less likely to start sexual activity early than those who had poor parental monitoring. This is consistent with studies done in Awi Zone [11], Kenya [32], and Nekemte [25]. Effective parental monitoring of children’s behavior, attitude, and values has a significant role in reducing poor decision making on sexual and reproductive life [28].

Furthermore, in this study, students who were exposed to pornographic materials were more likely to initiate sexual activity before the age of 18 years. This finding agrees with those of other studies conducted in Shire-Endasellassie town [18], Alamata [12], and Bahir Dar [17], Ethiopia. This could be due to the fact that pornographic materials can stimulate psychological and mental sexual desire and empress to experiment what has been observed. The impulsive nature of pornographic materials lead to erotic sexual stimulation or early sexual practice [33, 34].

## Limitations of the study

Since sexual practice has private, intimate, and sensitive nature in the society, there might be an underreporting of some behaviours. However, the principal investigator used self-administered questionnaire so as to maintain privacy and informed participants about the purpose of the study and the importance of telling the truth.

## Conclusion

A significant proportion of students had sexual practice before the age of 18 years. Not attending religious programs, peer pressure, smoking cigarettes, poor parental monitoring, and watching/reading pornographic materials were significantly associated with early sexual initiation. The authors recommended increasing awareness and educating students to delay sexual practice, avoid substance use, and viewing or reading pornographic materials. The recommendation to families is to monitor their children and support them to have good school connectedness.

## Additional file

Additional file 1: English version research questionnaire used to assess the factors associated with early sexual initiation among preparatory and high school youths in Woldia town, northeast Ethiopia: A cross-sectional study. (DOCX 30 kb)

## Abbreviations

AOR: Adjusted Odds Ratio
CI: Confidence Interval
COR: Crude Odds Ratio
EDHS: Ethiopian Demographic and Health Survey
IQR: Inter Quartile Range
PCA: Principal Component Analysis
SPSS: Statistical Package for Social Sciences
SRH: Sexual and Reproductive Health
UNICEF: United Nations Children’s Fund
USA: United States of America
