# Supplementary material for: Factors associated with early sexual initiation among preparatory and high school youths in Woldia town, northeast Ethiopia: a cross-sectional study
Source: BMC Public Health. 2019 Apr 4;19:378. doi: 10.1186/s12889-019-6682-8 (PMC6450012; doi:10.1186/s12889-019-6682-8)
Supplement: Supplementary file 1 — English version research questionnaire used to assess the factors associated with early sexual initiation among preparatory and high school youths in Woldia town, northeast Ethiopia: A cross-sectional study. (DOCX 30 kb) [file 12889_2019_6682_MOESM1_ESM.docx]

| **Instruction: Please indicate your answer by circling the number of your choice or by writing your response in the space provided accordingly**  **Part one: Socio-demographic and economic characteristics of respondents** | | | | | | | | |
| --- | --- | --- | --- | --- | --- | --- | --- | --- |
| **s.no** | **Questions** | | **Answer** | | | | | |
| 101 | Sex | | 1. Male 2. Female | | | | | |
| 102 | Age | | ________years | | | | | |
| 103 | Educational status | | 1. grade 9^th^ 2. grade 10^th^ 3. grade 11^th^ 4. grade 12^th^ | | | | | |
| 104 | Residence | | 1. Urban 2. Rural | | | | | |
| 105 | What is your ethnicity? | | 1. Amhara 2. Tigre 3. Oromo 4. Others specify____________ | | | | | |
| 106 | What is your religion? | | 1. Orthodox Christian 2. Muslim 3. Protestant 4. Catholic 5. Other (specify) _____________ | | | | | |
| 107 | Have you attending religious institution? | | 1. Yes 2. No --------------**skip to Q.109** | | | | | |
| 108 | How often do you visit the religious institution? | | 1. Every day 2. Once a week 3. Once a month 4. Once a year 5. Never attend 6. Other specify___________ | | | | | |
| 109 | What is your marital status? | | 1. Single 2. Married 3. Other specify__________ | | | | | |
| 110 | Living arrangement | | 1. With both parents 2. With mother only 3. With father only 4. With sister and/or brother 5. with friends 6. With relatives 7. living alone 8. With spouse /sexual partner 9. Others/specify______________ | | | | | |
| **Part II: Questions related to Parent, peer and school conditions** | | | | | | | | |
| 201 | Is your father alive? | | 1. Yes 2. No -----------------**skip to Q. 204** | | | | | |
| 202 | What is your father educational status? | | 1. Unable to read and write 2. Able to read and write 3. Elementary school 4. High school 5. Preparatory school 6. Collage and higher education | | | | | |
| 203 | What is your father employment status currently? | | 1. Civil servant 2. Private employer 3. Merchant 4. Daily laborer 5. Farmer 6. Others(specify__________ | | | | | |
| 204 | Is your mother alive? | | 1. Yes 2. No ----------------**skip to Q. 207** | | | | | |
| 205 | What is your mother’s educational status? | | 1. Unable to read and write 2. Able to read and write 3. Elementary school(1-8^th^) 4. High school(9-10^th^) 5. Preparatory school(11-12^th^) 6. Collage or higher education | | | | | |
| 206 | What is your mother occupation? | | 1. House wife 2. Civil servant employer 3. Private employer 4. Merchant 5. Daily laborer 6. Farmer 7. Others(specify)______________ | | | | | |
| 207 | Total no. of families living in the household | | ____________(in number) | | | | | |
| **Parent monitoring and communication with youth on sexual issue** | | | | | | | | |
| 208 | How often do your parents try to know who are your friends? | | 1. Never 2. Sometimes 3. Usually 4. Always | | | | | |
| 209 | Do your parents know where you are outside of home or school? | | 1. Never 2. Sometimes 3. Usually 4. Always | | | | | |
| 210 | Do your parents know with whom you are outside home or school? | | 1. Never 2. Sometimes 3. Usually 4. Always | | | | | |
| 211 | Have you ever discussed about sexual and reproductive health issue with your parents? | | 1. Yes 2. No ----------------**skip to Q. 213** | | | | | |
| 212 | **If your answer is yes for Q 211,** on which sexual and reproductive health issues you have discussed with your parents? **(multiple answer possible)** | | 1. Body change during puberty/menstruation 2. How to avoid getting pregnancy 3. Relationship with opposite sex 4. Unwanted pregnancy and/or abortion 5. Sexually transmitted diseases and/or HIV/AIDS 6. Other specify-------------------------- | | | | | |
| **Implication of peer influence to have sexual intercourse** | | | | | | | | |
| 213 | Do you have pressure from your friend(s) to have sexual intercourse? | | 1. Yes 2. No | | | | | |
| **Life skills training related question** | | | | | | | | |
| 214 | Have you ever taken life skills training before? | | 1. Yes 2. No | | | | | |
| **Questions related to youth’s perception on school connectedness** | | | | | | | | |
| **s.no** | **Youth School**  **Connectedness** | **Strongly agree** | **Agree** | **Not sure** | | | **Disagree** | **Strongly Disagree** |
| 215 | Do you feel close to people who are at school? | 5 | 4 | 3 | | | 2 | 1 |
| 216 | Do you feel happy to be at this school? | 5 | 4 | 3 | | | 2 | 1 |
| 217 | Do you feel as if you are a part of this school? | 5 | 4 | 3 | | | 2 | 1 |
| 218 | Do school teachers treat students fairly at this school? | 5 | 4 | 3 | | | 2 | 1 |
| 219 | Do you feel safe being at this school? | 5 | 4 | 3 | | | 2 | 1 |
| **Part III: Questions related to HIV/AIDS** | | | | | | | | |
| 301 | Have you ever heard of an illness called HIV/AIDS? | | | | | 1. Yes 2. No | | **Skip to Q. 401** |
| 302 | Can people reduce their chances of getting the HIV/AIDS virus by having just one uninfected sex partner who has no other sex partners? | | | | | 1. Yes 2. No 3. Don’t know | |  |
| 303 | Can people get the HIV/AIDS virus from mosquito bites? | | | | | 1. Yes 2. No 3. Don’t know | |  |
| 304 | Can people reduce their chance of getting the AIDS virus by using a condom every time they have sex? | | | | | 1. Yes 2. No 3. Don’t know | |  |
| 305 | Can people get the AIDS virus by sharing food with a person who has AIDS? | | | | | 1. Yes 2. No 3. Don’t know | |  |
| 306 | Can people reduce their chance of getting the AIDS virus by abstaining from sexual intercourse? | | | | | 1. Yes 2. No 3. Don’t know | |  |
| 307 | Can people get the AIDS virus because of witchcraft, God's curse, or other supernatural means? | | | | | 1. Yes 2. No 3. Don’t know | |  |
| 308 | Is it possible for a healthy-looking person to have the AIDS virus? | | | | | 1. Yes 2. No 3. Don’t know | |  |
| 309 | Can people get the AIDS virus by sharing sharp materials such as razors/blades or through injection with non-sterilized needles? | | | | | 1. Yes 2. No 3. Don’t know | |  |
| **Part IV: Part three: Questions related risk related behaviors of school youths** | | | | | | | | |
| 301 | Do you drink alcoholic beverages like Tella, Tej, beer, arekie, and the like? | | | | 1. Yes 2. No | | | |
| 302 | Do you smoke cigarettes? | | | | 1. Yes 2. No | | | |
| 303 | Do you chew khat? | | | | 1. Yes 2. No | | | |
| 304 | Do you smoke shisha? | | | | 1. Yes 2. No | | | |

| **Part IV: Questions related to sexual behavior of school youths** | | | | |
| --- | --- | --- | --- | --- |
| 401 | | Have you ever had sexual intercourse? | 1. Yes 2. No ---------**skip to Q. 501** | |
| 402 | | If your answer is yes for **Q. 401**, How old were you when you had sexual intercourse for the very first time? | **Age_________** in years | |
| 403 | | At the time you had first sexual intercourse, what was your relationship with your partner? | 1. With a steady boy/girl friend 2. With casual boy/girl friend 3. With husband /wife 4. With family member 5. With my teacher 6. With commercial sex worker 7. Other, specify_______________ | |
| 404 | | What are the factors that encouraged you for the first sex? (you can answer more than one) | 1. I get married 2. Fell in love 3. Peer influence 4. Personal desire 5. Influence of khat 6. Influence alcohol 7. Coerced/rape 8. Convinced with money or gift 9. Initiated by pornographic materials 10. Other specify----------------------- | |
| 405 | | How much older or younger was the person with whom you had first sex? | 1. She/he was similar age with me 2. More than 10 years 3. 5-10 years older 4. Less than 5 years older 5. younger 6. Do not know | |
| 406 | | Have you used condom during your first sex? | 1. Yes ----------**skip to 408** 2. No | |
| 407 | | How many sexual partner(s) have you had so far? | 1. Three and more 2. Two 3. One | |
| 408 | | Did you have sexual intercourse in the last 12 months? | 1. Yes 2. No--------------**skip to Q. 501** | |
| **Part five: questions related to pornographic Material ( sex films, newspaper, magazine, books, photographs) in the** **last 12 months** | | | | |
| 501 | | Have you ever viewed/read/sea pornographic material (sex films, newspaper, magazine, books, photographs which arose sexual desire)? | | 1. Yes 2. No **--------skip to 601** |
| **Part Six: Household wealth index related questions** | | | | |
| 601 | Owner ship of the house | | | 1. Private 2. Rented from individual 3. Others(specify)____________ |
| 602 | Number of rooms in the household are used for sleeping | | | ____________in number |
| 603 | Main material of the dwelling floor | | | 1. Earth / Sand 2. Dung 3. Cement 4. Bamboo 5. Carpet 6. Others(specify)**____________** |
| 604 | Main material of the roof | | | 1. Iron corrugated sheet 2. Wood 3. Thatch 4. Bamboo 5. Others (specify)__________ |
| 605 | Main material of the exterior walls | | | 1. Stone with mud 2. Wood with mud 3. Stone with cement 4. Others specify --------------- |
| 606 | Type of fuel mainly used for household cooking | | | 1. Electricity 2. Charcoal 3. Wood 4. Animal dung 5. Others (specify)___________ |
| 607 | Is the cooking usually done in the house, in a separate building, or outdoors? | | | 1. In a separate room used as kitchen 2. Elsewhere in the house 3. In a separate building 4. Outdoors 5. Other (specify)___________ |
| 608 | Does any member of the household own any land that can be used for agriculture? | | | 1. Yes 2. No |
| 609 | Ownership of the agricultural land | | | 1. Own, in hectares/gemed____ 2. Rent, in hectares/gemed____ |
| 610 | Annual total agricultural products(includes all items) | | | ______________kuintal |
| 611 | Does your house hold have?   1. Electricity? | | | \| Yes \| No \| \| --- \| --- \|   1 2 |
|  | 1. A Radio? | | | 1 2 |
|  | 1. A Television? | | | 1 2 |
|  | 1. A Non-mobile telephone | | | 1 2 |
|  | 1. A Refrigerator? | | | 1 2 |
|  | 1. Table? | | | 1 2 |
|  | 1. Chair? | | | 1 2 |
|  | 1. A bed with cotton/spring mattress | | | 1 2 |
| 612 | Does any member of your household own?   1. A watch? 2. A mobile phone? 3. A bicycle? 4. A Bajaj? 5. Animal drawn cart? 6. Car? | | | \| Yes \| No \| \| --- \| --- \| \| 1 \| 2 \| \| 1 \| 2 \| \| 1 \| 2 \| \| 1 \| 2 \| \| 1 \| 2 \| \| 1 \| 2 \| |
| 613 | Does this household own any livestock, herds, other farm animals, or poultry? | | | 1. Yes 2. No |
| 614 | How many of the following animals does the household have? (if the household does not have the listed animal use 999) | | |  |
|  | 1. Cattle, milk cows, bulls? | | | _________in number |
|  | 1. Horses, Donkeys, or mules? | | | _________in number |
|  | 1. Goats? | | | _________in number |
|  | 1. Sheep? | | | _________in number |
|  | 1. Chickens? | | | _________in number |
|  | 1. Beehives? | | | _________in number |
| 615 | Does any member of this household have a bank account? | | | 1. Yes 2. No |

That is the end of our questionnaire. Thank u very much for taking time to answer these questions. We appreciate your help!!!!
